# Urban seismic monitoring in Brasília, Brazil

**DOI:** 10.1371/journal.pone.0253610

**Published:** 2021-08-05

**Authors:** Susanne Taina Ramalho Maciel, Marcelo Peres Rocha, Martin Schimmel

**Affiliations:** 1 Faculdade UnB Planaltina/Exatas, Universidade de Brasilia, Brasilia, DF, Brazil; 2 Instituto de Geociencias/Observatorio Sismológico, Universidade de Brasilia, Brasilia, DF, Brazil; 3 Geosciences Barcelona (GEO3BCN - CSIC), Barcelona, Spain; Universidade Estadual de Maringa, BRAZIL

## Abstract

Urban seismology has gained scientific interest with the development of seismic ambient noise monitoring techniques and also for being a useful tool to connect society with the Earth sciences. The interpretation of the sources of seismic records generated by sporting events, traffic, or huge agglomerations arouses the population’s curiosity and opens up a range of possibilities for new applications of seismology, especially in the area of urban monitoring. In this contribution, we present the analysis of seismic records from a station in the city of Brasilia during unusual episodes of silencing and noisy periods. Usually, cultural noise is observed in high-fequency bands. We showed in our analysis that cultural noise can also be observed in the low-frequency band, when high-frequency signal is attenuated. As examples of noisy periods, we have that of the Soccer World Cup in Brazil in 2014, where changes in noise are related to celebrations of goals and the party held by FIFA in the city, and the political manifestations in the period of the Impeachment trial in 2016, which reached the concentration of about 300,000 protesters. The two most characteristic periods of seismic silence have been the quarantine due to the COVID-19 pandemic in 2020, and the trucker strike that occurred across the country in 2018, both drastically reducing the movement of people in the city.

## Introduction

Seismometers are traditionally installed for earthquake monitoring. Nevertheless, there is a variety of sources to which seismic signals can be assigned, including a set of complex interactions between ocean swell and atmospheric pressure (e.g. [1, 2]), winds and storms (e.g. [3]), and also patterns of human activities (e.g. [4]). It is often strategic to install seismometers away from urban centers, where anthropogenic noise can degrade the ability to detect transient signals. However, monitoring of ground motion in urban environments have an important role in the maximization of the spatial coverage of seismic networks, warning systems of local geological hazards, and also for education purposes (e.g. [5–7]). Urban seismology is a relatively new research field, related to the concept of citizen seismology, and it takes advantage of that seismographic stations can record mobility patterns in populated areas [4]. Thus, seismology can track the recent cultural and political history, with peaks of amplitudes during unusual people’s movement. In the last decade, urban seismology has appeared in many applications [8]. It is an essential tool to create microzonation maps. Still, it is also relevant for soil mapping [9], monitoring of traffic or economic growth [10], or even cultural observations, such as the identification of mass movement of people in soccer tournaments, concerts, or dancing events [4, 11, 12]. Therefore, the characterization of urban seismic sources is a substantial contribution to seismological literature. We assessed the seismic records from a broad-band station during remarkable events in Brasilia, Brazil, to delineate and characterize urban seismic sources in two different situations: big crowds of people agglomeration and unusual occurrences of an absence of anthropogenic noise.

People agglomerations trigger footquakes that can be detected by urban seismometers. We identified events of people gathering at more than 30 km distance from the station. An example of a footquake is associated with the Soccer World Cup Games, in 2014, when Brasília hosted four matches, including Brazil versus Cameroon dispute, that gathered almost 70.000 soccer fans. A second example is related to the protests resulting from the impeachment process of ex-president Dilma Roussef, also in 2016. The streets of all the country’s capitals were filled with people who were for and against the Dilma government, in a series of protests that started in 2015. In April 2016, the congressional vote for the removal of the then president gathered more than 300.000 protesters at the Esplanada dos Ministerios, located in the central region of Brasilia, approximately 30 km from the station being used. Big crowds’ movement resulting from memorial gatherings, or political demonstrations, result in seismic spikes at specific bands visible in spectrograms to the naked eye.

In contrast, silent periods also revealed important details about urban seismic sources. On January 30, 2020, the World Health Organization (WHO) declared that the outbreak of COVID-19, constituted a Public Health Emergency of International Importance (the highest level of WHO warning). Subsequently, on March 11 2020, the WHO declared COVID-19 as a pandemic. Since it was a new type of virus and we had no specific therapeutics or vaccines with scientifically proven efficacy, it was required to take alternative measures to reduce the transmission intensity and consequentially not to overload the health system worldwide. Among these measures, one of the most traditional and efficient ways used by public health was to interrupt transmission by separating people, applied in various ways, depending on the pandemic stage or the intensity of its consequences [13]. Since the beginning of the pandemic, most governments in all countries have implemented social distancing, at different times and with different strength.

The outbreak of the COVID-19 pandemic, and the consequent orientation towards physical distancing, permitted seismologists to observe an unprecedented decay on seismic noise amplitudes that moved into the focus of recent seismological studies [14–24]. After the many countries have declared social distance measures, several news agencies and scientific communication channels started to report the reduction of seismic noise in different seismographic stations around the world [16, 25]. The countries that have passed through isolation measures registered a drop of 50% (global median) in urban seismic noise around the implementation of the decrees for schools and business closures [17]. The social distance measures had a direct impact on the economic activities of the cities where they were implemented. Some works show the relationship between the variation in the amplitude of seismic noise and socio-economic indicators [10, 26].

The COVID-19 pandemic reached Brazil later than other countries, but was considered one of the worst epicenters of the disease, with more than 14 million confirmed cases at the beginning of April 2021. The capital, Brasilia, located in the Distrito Federal, had its first case recorded on March 07, 2020. The capital’s government established the first decree for school closures on March 19, 2020, and the second decree establishing business closures on April 10, 2020. This first quarantine period was the most respected by the population throughout the pandemic and it was when the mobility index was the lowest registered in Brasília according to Google’s Mobility Index [27].

In addition to the silence period caused by the COVID-19 pandemic, in Brazil, another similar silence period occurred in 2018, during the biggest truckers’ strike the country has ever had. The so-called “Diesel crisis” was a stoppage of autonomous truck drivers, with national extension, that started on May 21 and ended with the intervention of forces from the brazilian army and the federal police on May 30. The truckers’ strike caused a shortage of food and medicines across the country, as well as a shortage and increase in the value of fuels. The lack of fuel in the cities caused the movement in the streets to decrease drastically. School classes were suspended, bus fleets were reduced, and flights were canceled. These different patterns of low-noise periods provided a unique opportunity to analyze the full spectrum of seismogenic behavior.

We focused our seismic noise analysis on one single station (BDFB), installed inside the Brasilia urban perimeter since 1993 [28]. We analyzed the PPSD (Probabilistic Power Spectrum Density) of the seismograms at various intervals of time between 2013 and 2020, and our findings revealed that even with a single station (BDFB), installed at about 30 km from the city center, urban noise sources were recorded and could be monitored.

## Data and methods

**Fig 1:**
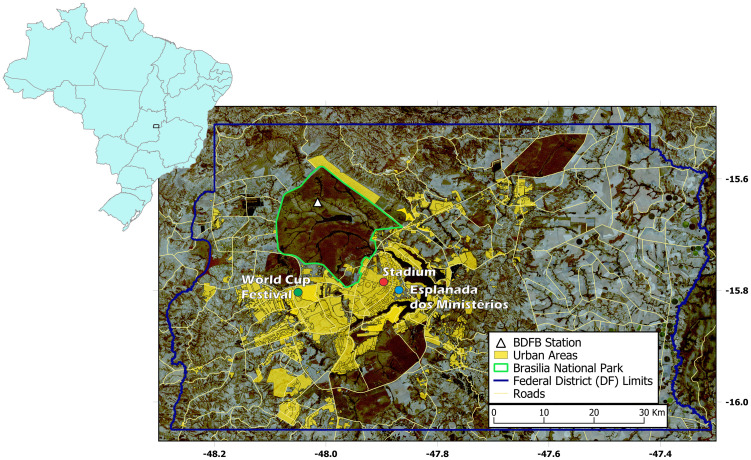
BDFB station. All seismic signals in this paper were recorded by BDFB station, located within the National Park, in Brasilia. The station is located 30 km away from the center of the city, and 10 km away from highways. (Background image available at Landsat [29]).

The BDFB station is part of the Comprehensive Nuclear-Test-Ban Treaty Organization (CTBTO) agreement [28], and is installed inside the National Park in Brasilia (Fig 1). It is a broad-band, borehole station installed at 100 m depth, recording at 100 samples per second (sps). The city noise sources are at least 10 km away from the station and are mainly driven by the highway surrounding the National Park. The city center is located 30 km away SE from the station (Fig 1).

Pre-processing of raw seismic data consisted in removing the instrument response to displacement. The power spectral densities (PSD) were calculated for every 30min segment with an overlap of 15 min. Then we extracted the RMS (root mean square) displacement for each segment, and determined the RMS median values for all segments between 07 AM and 07 PM local time. These steps were performed with the codes provided by Thomas Lecocq (https://github.com/ThomasLecocq/SeismoRMS—last access May/2020).

The processed data was analyzed and we could observe trends and peaks on the seismic data whose interpretations are presented in the following sections. For the case of COVID-19 lockdowns, we used Google Mobility Reports [27] to validate our interpretation. Data and codes are available at https://github.com/sutaina/brasiliaNoise.git.

Google Mobility Reports [27] are a set of time-series based on movement trends over time registered by smartphones, and were made public as a result from the demand for information on the degree of physical isolation of specific regions during COVID-19 lockdowns. These reports were useful to correlate the drop of amplitudes in the seismic noise with the sudden change in the movement of people in the city. Both data, seismic noise and mobility reports, presented visual changes of pattern during lockdown phases in Brasilia. A way to determine numerically when these changes happen is using change-point analysis (CPA).

CPA is an approach to search points in time series where the statistical properties of the data significantly changes. CPA has already been used for seismic signal analysis. For example, [30] introduced CPA in seismic interferometry to identify preeruptive phases before volcanic eruption. Change-point detection methods are essentially the combination of three main elements: a cost function, a search method, and a constraint on the number of changes to detect. It can be posed as an optimization problem with a fixed number or an unkown number of change-points. In our case, where the number of change-points are unknown, we used the algorithm Pelt (“Pruned Exact Linear Time”) [31] as the search method. The cost function is a measure of the uniformity of a signal. In other words, a change-point will be detected on an interval of the time series whenever the cost function is large, indicating that that part of the signal is not “uniform.” The uniformity of a time series, though, is a relative concept encoded by the cost function choice. In our case, we used the RBF cost function, which is a non-parametric approach based on a gaussian kernel estimation [32]. It means that it can detect distribution changes, rather than shifts in standard statistical measures, such as mean, or mode, etc.

We used the rupture library, designed to perform CPA within Python [33]. The rupture library provides several implementations for cost functions and search methods. We used the Pelt search method with a radial basis function model, which assumes that the number of change-points is unknown. With this configuration, the only parameter that is demanded is the penalty value in the prediction step. Convenently, the library also provides some metrics calculations. We choose the penalty value by making an iterative search on a set of penalty values. For each value, we evaluated the precision and recall metrics for each segmentation. The penalty value chosen was the one that optimized the precision-recall trade-off (2 in our case—see S1 Fig).

## Seismic noise sources overview

A broad-band seismic station is continuously recording noise from distinct physical phenomena, such as ocean waves, traffic, wind waves, or machinery. The waves emited by each noise source appear in distinct bands of the seismic spectra. Low frequency events (0.002–0.03 Hz), also known as “seismic hum”, are caused by ocean infra gravity waves due to atmospheric perturbations [34, 35]. At intermediate frequency bands (0.03–1 Hz), it is common to observe microseisms that usually stand out in magnitude from other parts of the spectrum [36]. Wind contributes to events observed in the 0.6–12 Hz band, and rivers and other natural sources have been documented to appear at the high-frequency band (above 1 Hz) [3]. Urban noise, or cultural noise, generally occurs at the high-frequency band (1–10 Hz) and includes seismic energy excited by trains and subways [37, 38], machinery, cars, or people movement [10, 11, 39]. Human activity is mainly observed for frequencies above 1 Hz, although some urban events might be recorded in lower frequency bands. For example, the subway regular time activity cycle also produces signals that are registered between 0.008 and 0.05 Hz [4].

In the recent years, the deployment of seismic networks in urban environments are being used for several applications on the investigation of the uppermost crust using ambient seismic noise. Site characterization applications are usually done with a high number of stations [3], although single stations ambient noise records are also used for retrieving the Green’s function through autocorrelation [40]. Single stations in urban areas are also being used for scientific divulgation and educational purposes [4, 5].

## Peculiar urban noise sources in and around Brasilia

### Footquakes during the Soccer World Cup

Ground motion triggered by people’s movement during music concerts or soccer matches have often been reported (e.g. [11, 12, 39, 41]), usually presenting significant impact on social networks. Footquakes during sport events are typically recorded by seismic instruments that are installed nearby the stadium [4], and in some cases, at the stadium, as is the case of the seismic monitoring network at the Seahawks stadium (https://www.pnsn.org/seahawks). The proximity of the seismometer with the footquake sources mitigates the effects of attenuation of the high frequency amplitudes, which provides nice seismogram records from details of the matches.

**Fig 2:**
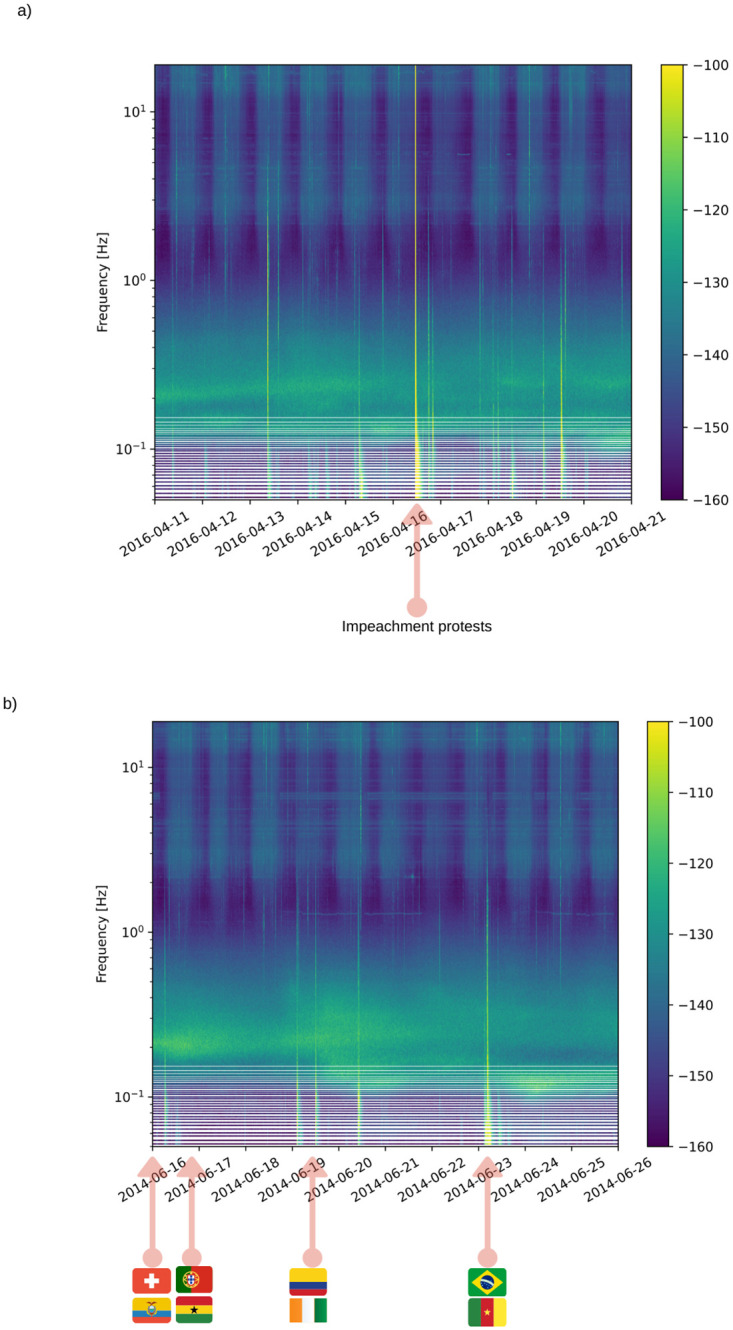
Spectrograms of World Cup in Brazil and Dilma Roussef protests. a) Spectrogram of BDFB station during the impeachment process. The event highlighted on April 17, 2016, marks the 300 thousand people protest at the Esplanada of the Ministries. b) Spectrogram of BDFB station during Soccer World Cup matches. The arrows show four matches in 2014 (Switzerland vs. Equator—June 15, Portugal vs. Gana—June 16, Colombia vs. Costa do Marfim—June 19 and Cameroon vs. Brazil—June 23), recorded mainly at the low-frequency band, below 1.0 Hz.

**Fig 3:**
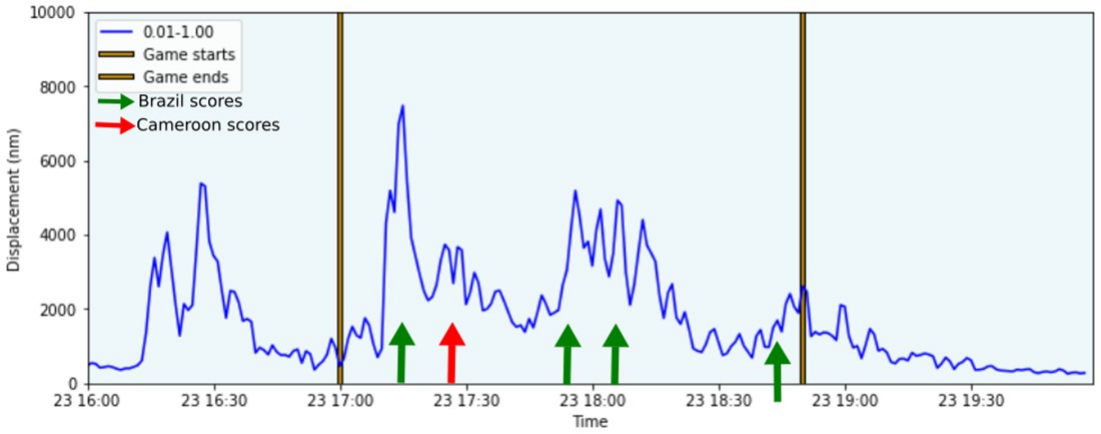
Cameroon vs Brazil scores and the recorded noise. Absolute ground displacement recorded in the frequency band between 0.01–1.0 Hz during World Cup match. Arrows indicate each score moment.

The BDFB station, on the other hand, is located 30 km away from Brasilia main stadium, Mane Garrincha (Fig 1). We analyzed the records during the World Cup games, in 2014, that gathered up to 70,000 people at the stadium. The spectrogram of the signals recorded during the World Cup (Fig 2a) revealed higher energy at the days with matches in Brasilia (namely Switzerland vs. Equator—June 15, Portugal vs. Gana—June 16, Colombia vs. Costa do Marfim—June 19 and Cameroon vs. Brazil—June 23), recorded mainly at the low-frequency band, below 1.0 Hz. Single station signals do not allow doubtful events to be checked, but we noticed that higher energy bursts matched the timings of goal celebrations (Fig 3 for an example). We also noted other similar features aside soccer matches, that may be associated with the festive program linked to the World Cup, which included daily concerts and transmission of the games in a park located 30 km from the BDFB station (see Fig 1). Soccer footquakes have been registered for frequencies between 1–10 Hz (e.g. [39, 41]). These registers may differ from ours either from the distance between stadium and station, as from local attenuation. Each match brought together around 70,000 people in the stadium, but Brazil and Cameroon match translated into higher amplitudes, perhaps because, in general, people celebrate goals in more parts of the city, in addition to the stadium. For this specific match, we analyzed the absolute ground displacement for the frequency band between 0.01–1.0 Hz, and we noticed that peaks of amplitudes match the scores’ times (Fig 3). The big peak around 16h30 is probably related to the concert that took place before the match, in the park where the festive program was happening. We also suspected that the transmission of the match at the park had a delay, what might explain the repetition of the scores peaks.

### Footquakes during political protests

**Fig 4:**
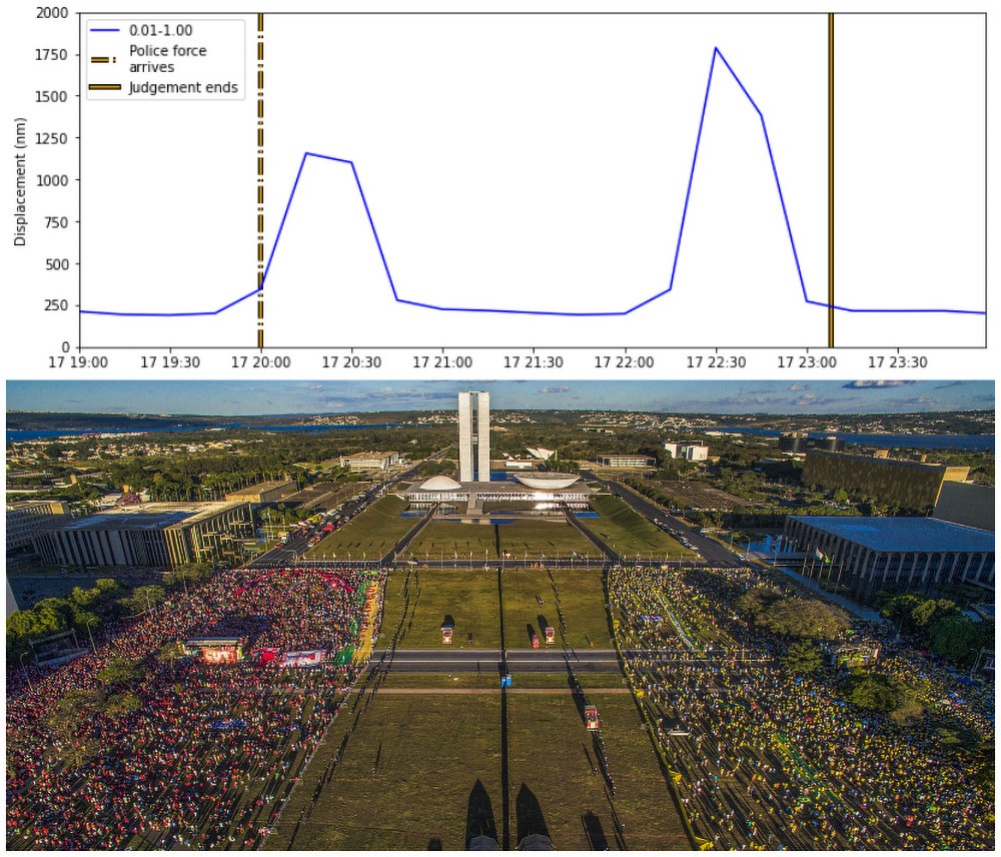
Ambient noise for Dilma Roussef’s impeachment judgement. Top: 0.01—1.0 Hz time series from when the Chamber of Deputies judged the impeachment. Bottom: aerial view from the protest. The city governor installed a temporary wall in the middle of the Esplanada do Ministerios to avoid conflicts between protesters from different political currents, dividing the groups against and in favor of the referral of the case to the Senate (picture by Ricardo Stuckert/FotosPublicas).

Another footquake event visible in the low-frequency band was the protests in front of the Chamber of Deputies on April 17, when Dilma Roussef impeachment was judged by the congress (Fig 2b). Social movements and the press announced that the protest gathered more than 300,000 people. The event appeared in the low-frequency band, indicated by a red arrow in Fig 2b. Other spikes were visible in the same spectral band, probably due to minor manifestations that happened all along the week. A local journal registered that around 8 PM, more than 4 thousand police officers arrived at the scene, and this time coincided with a peak observed on the signal filtered for the 0.01–1.0 Hz band (Fig 4). The decisive vote took place around 11 PM, and we interpreted the peak amplitude between 10 PM and 11 PM (Fig 4) as the movement and agitation caused in the final minutes of the trial when the decision was about to be made. There are no records of conflicts after the trial, which was also observed with the absence of peaks in the seismic data after 11 PM.

### Silent period during COVID-19 lockdowns

Periods of low noise levels are of seismological interest for many reasons. For example, some distant small magnitude events that are usually disguised by noise might appear at a low noise period. Silent periods are also useful for identifying noise sources that vanish when some specific activity is suddenly stopped. We showed the amplitude decay on the records from two silent periods in Brasilia: COVID-19 quarantine in 2020 and a trucker strike in 2018.

**Fig 5:**
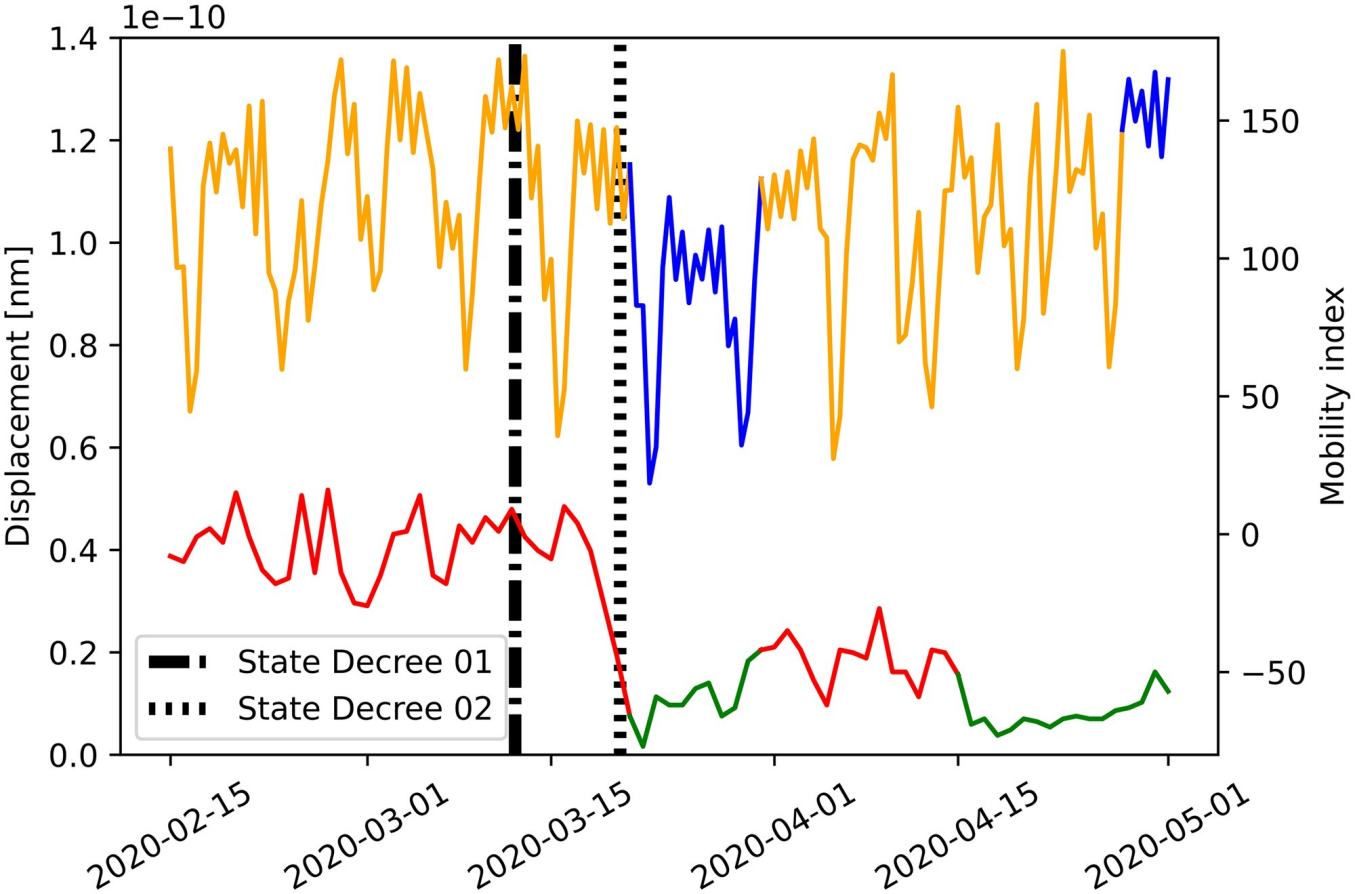
Seismic noise reduction during COVID-19 quarantine. CPA results for noise displacement amplitude at the station BDFB (Brasilia, Distrito Federal, Brazil) for the first four months of 2020, within the band 4–14 Hz, and Google Mobility index. The color changing depicts the results from change-point detection. The series in blue/orange represents the mean values of seismic noise displacement between 7 AM-7 PM. Black lines indicate time markers of COVID-19 state decrees—first: schools closure (dashed line), second state decree—business closure (dot-dash line). Google mobility index is in red/green, for a period of 2.5 months after the Carnival, including the COVID-19 quarentine in Brasilia.

The first physical distancing decree due to COVID-19 started a few weeks after Carnival. Carnival is a traditional Brazilian holiday that always starts on a Saturday (indicated in Fig 5 by a full red line) and lasts until the next Wednesday. The holiday might be related to the amplitudes drop observed between Saturday and Wednesday, in a pattern similar to the weekends’ typical energy decaying.

Fig 5 shows the energy drop of the recorded noise for the first weeks of physical distancing decrees in Brasilia, Brazil, due to COVID-19 pandemics. The countries that have passed through mobility restriction measures due to COVID-19 pandemics registered a drop up to 50% in the amplitude of the urban seismic noise [17]. For BDFB station, we observed a 20% decay on noise displacement amplitude after the second decree. The ground motion displacement signal was filtered for the band 4–14 Hz. The orange/blue line in Fig 5 shows the median value between 7 AM to 7 PM local time. To check if the decay of amplitudes after the decrease in people’s movement was caused by the physical distancing decrees, we compared the seismic noise time series with Google Mobility reports [27] (red/green line in Fig 5). We compared the changes of patterns from both the urban seismic noise variation and Google Mobility reports using Change-Point Analysis (CPA) (see Fig 5). CPA focuses on determining whether a change has taken place within a time-series [33]. CPA identified a changing point at the second decree in both time series. The forthcoming detected change-point was also coincident in both time series (Fig 5). F1-score for the estimated segmentation was 0.7, indicating a balanced match between the two sets of change-points (see S1 Fig for more details).

### Silent period during the truckers’ strike

**Fig 6:**
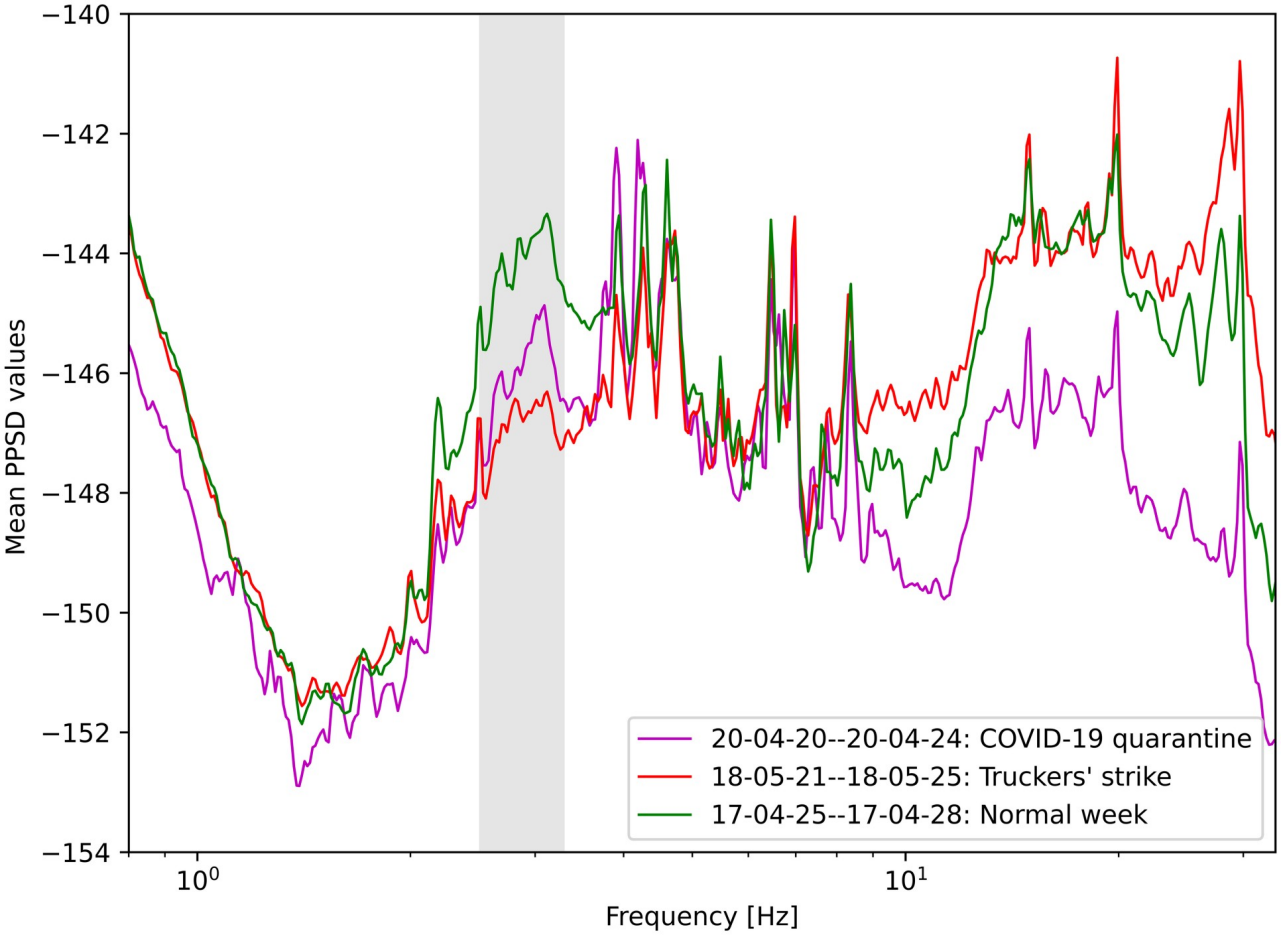
Mean PPSD values for truckers strike, COVID-19 physical distancing and a normal week. The mean PPSD values for ordinary days between April 25 and 28 in 2017 are shown in green. In 2018, May, a big truckers strike happened in Brazil, and the mean PPSD values for the registers between May 21 and 25 are shown in red. We showed the mean PPSD values in magenta for a quiet week during COVID-19 physical distancing decrees in Brasilia, between April 20 and 24, 2020. All periods are four days long and do not include holidays or weekends.

We also analyzed seismic data during a prominent national truckers’ strike that occurred between May 21 to 25, 2018, drastically decreasing the movement of the Brazilian highways. To investigate the impact of highways traffic on seismic records, we evaluated the mean PPSD values, which are useful to analyze the behavior of the signal for each frequency. Fig 6 shows the curves of mean PPSD for three different weeks registered by BDFB station, not including weekends or holidays. We showed the behavior of the mean PPSDs for frequencies between 1.0 and 100 Hz, where anthropogenic noise was predominantly observed. We included the PPSD mean values for the national truckers strike in red. The movement in highways also decreased during the first weeks of Brazilian quarantine due to COVID-19, so we included the period between 20th and 24th April 2020 (right after the second decree was established in Brasilia) shown in magenta (Fig 6). For comparison purposes, we included (in green) the mean PPSD values for an ordinary week in April 2017. PPSD shape for a physical distancing week is about the same as PPSD for a normal week, but with smaller amplitudes. The amplitudes drop during silent periods, however, varied with frequency. For frequencies higher than 10 Hz, the quarantine week presented lower amplitudes than the other weeks, as expected. But for the frequency band between 4.3 and 8.3 Hz, PPSD amplitudes for silent weeks and a normal week were similar. Possible noise sources not affected by COVID-19 quarantine may cause that. These peaks might be associated with some local noise sources, such as pump machinery from the reservoir located inside the park, but we need further investigation to conclude that.

[42, 43] reported the traffic noise spectra between 1 to 35 Hz. We observed the drop of the mean PPSD values in the frequencies between 2.5 to 3.3 Hz (grey bar in Fig 6), in the weeks when there was a sharp reduction in the movement of the highways that surround the BDFB station (truckers strike and COVID-19 quarantine). In this same period, the truckers’ strike PPSD reached lower values than COVID-19 PPSD. The city’s supply, and therefore the circulation of trucks, did not stop during the quarantine of COVID-19. On the other hand, during the truckers’ strike both, trucks and private cars, stopped circulating, since the city’s fuel supply comes through trucks, which were on strike. Our analysis suggests that the absence of heavy traffic is mostly perceived at this frequency band (Fig 6).

## Final evaluations

BDFB is a broad-band station, deployed at a calm and protected place (Fig 1, and operational since 1993. Data from this station has been used in several studies which means that this station is well tested and known. On the other hand, it is a single station, which means that there are no other independent measurements available from nearby places to check dubious events. In this sense, the ideal situation is to dispose of an urban seismic network which also permits to use array methodologies to characterize noise sources. Nevertheless, the usage of single stations remains important as often there exist no stable long-time deployments of urban networks.

Although BDFB is located at about 30 km from the city center and its soccer stadium, we showed the detection and identification of urban anthroprogenic noise sources. The signals created by the footquakes were observed in a wide frequency range, mainly at high frequencies as usual. We observed that the corresponding signals were also visible at lower frequencies than usually reported by studies based on seismic stations deployed close to the sources (e.g. [4, 5]). This fact might be explained by attenuation effects. Soil displacement varies with epicenter distance and attenuation, which in turn, varies with frequency. So for seismometers far away from the epicenter, the same event can have higher frequencies attenuated, but lower frequencies preserved.

The coronavirus lockdowns around the world and respective noise drop analysis made it possible to compare our results with a wide range of similar studies (e.g. [15–17, 22]). We did not see a difference as abrupt as those seen by other seismological observatories. Perhaps because culturally, the population has not respected the distancing guidelines so much, but more likely because, in addition to our station being far away, Brasilia is located on a plateau in central Brazil, which favors the formation of very thick and clayey soils, oxisols up to 100 meters thick, which significantly attenuate the surface wave.

Seismic noise data during the COVID-19 quarantine was interpreted and compared with the Google mobility report’s isolation index. CPA permitted to show that both time-series have coincident change-points which further are accord with the main COVID-19 decrees in 2020. It illustrates that BDFB records the gross mobility as testified by its response to the COVID-19 decree and measured with the Google mobility index and seismic motion. CPA is focused on finding changes in the underlying model of time-series. If two signals are monitoring related phenomena, then we expect that change-points from both series coincide. Our goal was to detect if the changes in mobility report and seismographic time-series were coincident, and to this end, we report that CPA has some merits for the task of comparison between signals, such as being easy to use, demands few parameters, and returns numerical evaluation metrics.

The discrimination of events that cause a reduction or increase in seismic noise in urban areas is not trivial. The examples presented are related to events that significantly altered the pattern of people’s behavior and therefore could be distinguished from the noise pattern on normal days. However, this type of study shows that urban monitoring using seismic data is possible, and that it can be improved. With this work, we seek to contribute to the design of urban arrays and the characterization of urban seismic sources. Silencing during the strike and physical distance allowed us to identify a narrower range of frequencies for which there is a significant change in the reduction in the volume of traffic compared to other studies [38]. For instance, circulating vehicles are usually recorded between 1–35 Hz (e.g. [42, 43]), but we also could detect the influence of vehicles in the 0.25–0.45 Hz frequency band, by analyzing the truckers’ strike and lockdown datasets. The analysis of two crowding episodes also lead to the same conclusion.

## Conclusion

Our results demonstrate that the use of a single station, distant from the noise sources, allows to detect the changes in the seismic noise, but that it is not the ideal condition to carry out an efficient monitoring of urban activity. The distance from the station leads to the attenuation of the high-frequencies, where anthropogenic noise is mostly observed. But our analysis shows that cultural noise is also registered in the low-frequency band.

The monitoring of urban seismic sources is vital for understanding the high frequencies of the seismic signal. We conclude that urban seismic arrays should ideally have at least two stations to allow more sophisticated signal processing steps. It is clear that more accurate analyzes could be made if there was an urban monitoring network available, with stations closer to the noise sources.

## Supporting information

S1 File Metrics for change-point analysis. (PDF)

S1 Fig CPA metrics. Precision and recall of the estimated segmentation for each penalty value chosen for comparison between seismic record during COVID-19 quarantine and Google mobility reports. In green, the Hausdorff metric, which measures the worst prediciton error.
(TIF)
