# Supplementary material for: Urban seismic monitoring in Brasília, Brazil
Source: PLoS One. 2021 Aug 5;16(8):e0253610. doi: 10.1371/journal.pone.0253610 (PMC8341495; doi:10.1371/journal.pone.0253610)
Supplement: S1 File — (PDF) [file pone.0253610.s001.pdf]

**S1 File. Metrics for change-point analysis.**

In the present problem, we can define precision as the fraction given by the number of change-points predicted for seismic displacement time-series that match change-points for mobility reports, divided by the number of change-points that are predicted for seismic displacement. Recall is the number of change-points predicted for seismic displacement time-series that match change-points for mobility reports, divided by the number of change-points predicted for mobility reports. Breakpoints are considered a match up to a user-defined margin of error. Over-segmentation of seismic time-series would cause its precision to be close to zero, and its recall close to one. Under-segmentation has the opposite effect. A good match between two sets of change-points can be evaluated by the F1-score, given by

$$F1 = 2\frac{P \times R}{P + R}, \tag{1}$$

where $P$ is precision and $R$ is recall. The best value for F1-score is one, and the worst case is 0.

The Hausdorff metric is equal to the greatest temporal distance between a change-point from the seismic time-series to the closest change-point from mobility index time-series (see S1 Fig).
