# Supplementary figures and images for: Urban seismic monitoring in Brasília, Brazil
Source: PLoS One. 2021 Aug 5;16(8):e0253610. doi: 10.1371/journal.pone.0253610 (PMC8341495; doi:10.1371/journal.pone.0253610)

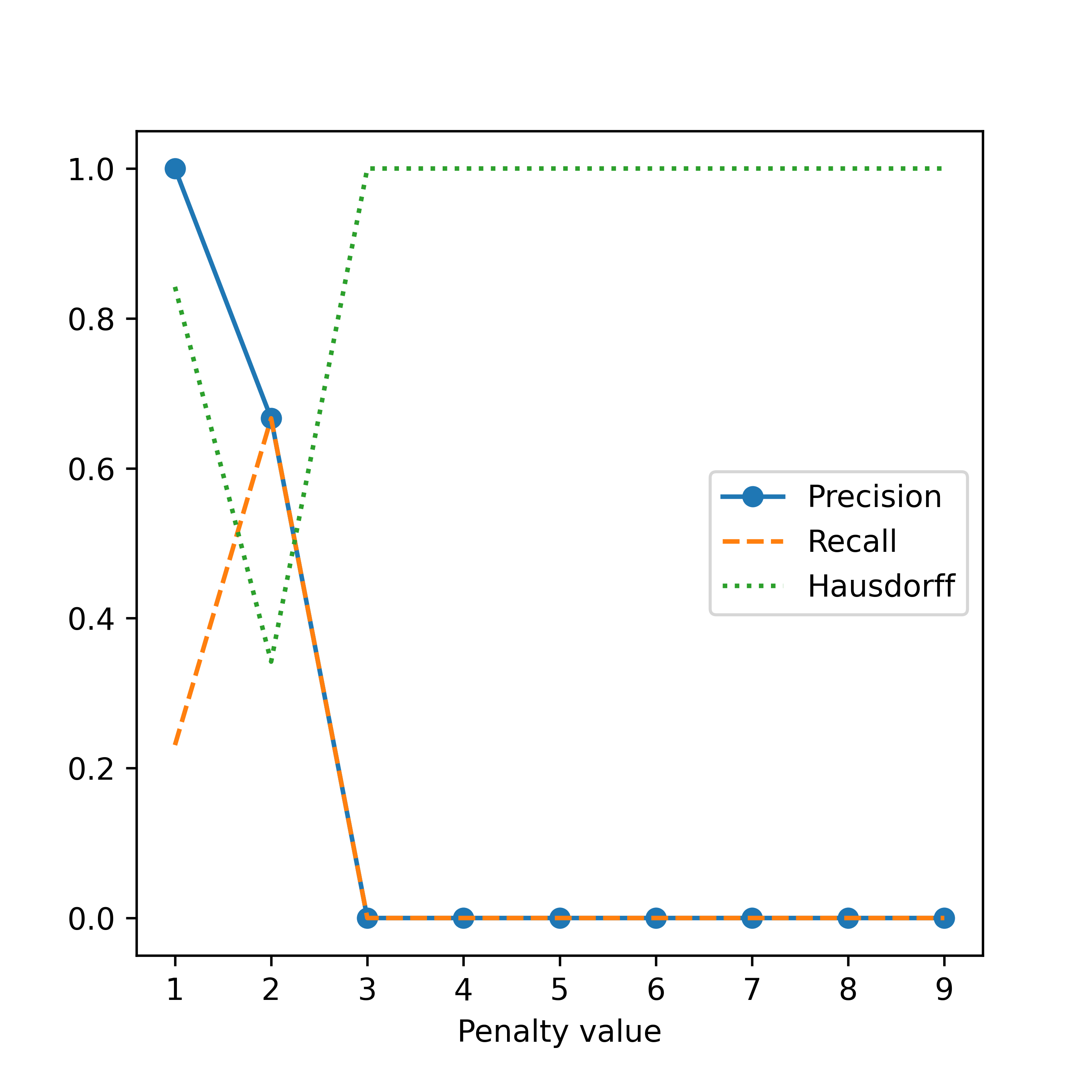

Supplement: S1 Fig — Precision and recall of the estimated segmentation for each penalty value chosen for comparison between seismic record during COVID-19 quarantine and Google mobility reports. In green, the Hausdorff metric, which measures the worst prediciton error. (TIF) [file pone.0253610.s002.tif]
